# Ventricular Tachycardia: A Treatment Comparison Study of the CyberKnife with Conventional Linear Accelerators

**DOI:** 10.7759/cureus.3445

**Published:** 2018-10-12

**Authors:** Georg A. Weidlich, Fred Hacker, David Bellezza, Patrick Maguire, Edward A Gardner

**Affiliations:** 1 Radiation Oncology, National Medical Physics and Dosimetry Company, Palo Alto, USA; 2 Radiation Oncology, Brigham and Women's Hospital, Boston, USA; 3 Medical Physics, St. Luke's Medical Center, Houston, USA; 4 Cardiac/thoracic/vascular Surgery, Cyberheart, Mountain View, USA; 5 Miscellaneous, Cyberheart, Mountain View, USA

**Keywords:** ventricular tachycardia, varian edge, varian truebeam, cardiac radiosurgery, cyberknife™, elekta infinity

## Abstract

The work described here compared the available technical solutions for the treatment of ventricular tachycardia with stereotactic body radiation therapy. Due to the complexity of target motion during cardiac and pulmonary motion as well as the several proximate radio-sensitive structures of the tracheobronchial tree and esophagogastrointestinal tract, four potential candidates for this treatment were identified: Accuray CyberKnife (Accuray Incorporated, Sunnyvale, California, United States), Varian TrueBeam (Varian Medical Systems, Palo Alto, California, USA), Elekta Infinity (Elekta, Stockholm, Sweden), and Varian Edge (Varian Medical Systems, Palo Alto, California, USA). All four treatment modalities were evaluated for their ability to deliver a conformal, homogeneous dose to most of the target volume, to spare nearby and distant critical and sensitive anatomical structures as well as for treatment efficiency. It was found that conventional linear accelerator technology was superior in their ability to spare distant critical structures and deliver treatments efficiently while the CyberKnife showed superiority in sparing nearby critical structures more aggressively by creating larger dose gradients at the periphery of the target volume. Both treatment modalities were similar in their ability to cover the entire target with the prescription dose, conform that dose to the target volume, and deliver a homogeneous dose.

## Introduction

Ventricular tachycardia (VT) is characterized by a rapid pathological beating of the ventricles of the heart [1]. It is a condition that is most commonly associated with damage to the heart induced by coronary artery disease. VT is a condition that can cause death or significant distress through other symptoms such as syncope, dizziness, palpitations, breathlessness, or chest pain. The standard of care includes the use of intracardiac defibrillators (ICDs) that shock the patient back to sinus rhythm at the onset of VT. However, quality of life suffers with frequent ICD shocks and survival is reduced when VT is frequent [2].

Drugs are often used in an attempt to prevent the occurrence of VT. However, drug treatment is frequently ineffective and is also associated with a significant risk of unpleasant side-effects. The failure of drug treatment, and its side-effects, in preventing VT and the desire to prevent recurrent shocks in patients with ICDs have led clinicians to promote the use of catheter ablation (CA) for VT. For patients who have failed drug treatment or who have ongoing issues with shocks, CA is an effective solution. Unlike ICD therapy, CA addresses the underlying cause of VT rather than its consequences. Accordingly, CA has been shown to reduce the recurrence of VT and the number of shocks delivered by ICDs. Unfortunately, CA also carries with it a substantial number of side-effects, including stroke, heart failure, and vascular and myocardial injury.

Due to the need for catheterization and the invasiveness of such procedures, several non-invasive techniques have been developed. Initial experience in radiosurgery for cardiac tumor was reported by Plowman et al. This case report demonstrated the feasibility and initial safety of a non-invasive approach to cardiac tissue ablation [3-4]. Given the accuracy and precision with which current technology allows the delivery of radiation to create fibrosis, block electrical conduction non-invasively, and employ advances in cardiac imaging, ‘cardiac radiosurgery’ has been developed to address this unmet clinical need [5]. One such method takes advantage of the ablative nature of large doses of radiation delivered over one to five treatment sessions using image-guidance for precise patient alignment and is commonly referred to as stereotactic body radiation therapy (SBRT) or stereotactic ablative radiation therapy (SABR). SBRT can deliver similar therapeutic results as mechanical, radiofrequency-based, or cryogenic ablation techniques and is, therefore, strongly considered by several research institutions and hospitals [6].

The objectives in treating VT with SBRT are the accurate delivery of the ablative dose to the target corresponding to the region causing aberrant conduction, conforming the treatment dose to the target region with a steep fall-off of radiation dose in order to spare identified proximal and distal critical anatomical structures, and optimizing temporal treatment efficiency. Furthermore, minimizing the total amount of monitor units (MU) is desired to decrease the compound leakage radiation that will determine the patient’s whole body dose, which may pose a long-term secondary malignancy risk for the patient. These objectives require the utilization of radiosurgery devices that can deliver the dose precisely to a small target moving with pulmonary and cardiac periodicity and the ability to create steep dose gradients at the target-to-critical-structure interface. The candidates for such a delivery technology selected for analysis in this modeling study are the Accuray CyberKnife (Accuray Incorporated, Sunnyvale, California, United States) with fixed collimators and with a multi-leaf collimator (MLC) [7], the Varian TrueBeam (Varian Medical Systems, Palo Alto, California, USA) [8], the Elekta Infinity (Elekta, Stockholm, Sweden), and the Varian Edge (Varian Medical Systems, Palo Alto, California, USA) [9] with a micro multi-leaf collimator. Special emphasis will also be placed on the unique imaging capabilities that this linear accelerator presents.

It is expected that the candidate treatment technologies have differing characteristics, making them more or less suitable to meet the stated treatment objectives for VT. Even though these treatment modalities were compared in the past from a technical or dosimetric perspective [10], it became apparent that the unique requirements of VT treatments with complex target motion, required precision, and anatomical configuration, necessitate a specific technology comparison for this application.

## Materials and methods

The origin of VT (and, therefore, the target of ablative therapy) is often a region of mixed scar caused by the occlusion of a coronary artery. However, the case considered in this study was the less-common, non-ischemic VT caused by aberrant tissue in the anterior basal portion of the heart. The degree of cardiac motion will be affected by the location of the target and the disease state of the patient. Apical locations will move more than basal locations in the heart while larger regions of existing scar reduce the contractility of the heart. In the case considered here, these effects were in opposition – a basal location (small motion) with very little scarring (large motion).

Each component of the target motion can be studied by employing four-dimensional computed tomography (4D CT) registered to the breathing cycle as well as a cardiac-gated 4D CT. The appropriate target expansion can be derived from such 4D CT studies and applied. While the CyberKnife can track a target volume that moves with pulmonary periodicity, linear accelerators (linacs) can gate the treatment delivery or treat a larger internal target volume (ITV) to account for the lung motion. Currently, both the CyberKnife as well as the linear accelerator treatment methods would include a separate target expansion to account for cardiac target motion derived from the cardiac-gated 4D CT. The appropriately expanded volume is the planning target volume (PTV), which will be used in the design and evaluation of the treatment plan.

Nine treatment plans were generated for the treatment of a VT target. The first and second plans were generated for the Accuray CyberKnife. The first used a fixed collimator with an Accuray Multiplan 5.1.2 treatment planning system while the second used a CyberKnife with multi-leaf Collimator (MLC) with an Accuray Precision 1.1.1 treatment planning system. The third, fourth, and fifth plans were generated for the Varian TrueBeam linear accelerator with RapidArc for the ITV approach, gating, and no expansion situations. The sixth plan was generated for an Elekta Infinity linear accelerator with volumetric arc therapy (VMAT) while the seventh, eighth, and ninth plans were generated for a Varian Edge. The third, fourth, fifth, and sixth plans were generated on a Philips Pinnacle 9.10 (Philips Radiation Oncology Systems, Fitchburg, WI, USA) treatment planning system while the seventh, eighth, and ninth plans were generated on Varian Eclipse.

**Figure 1:**
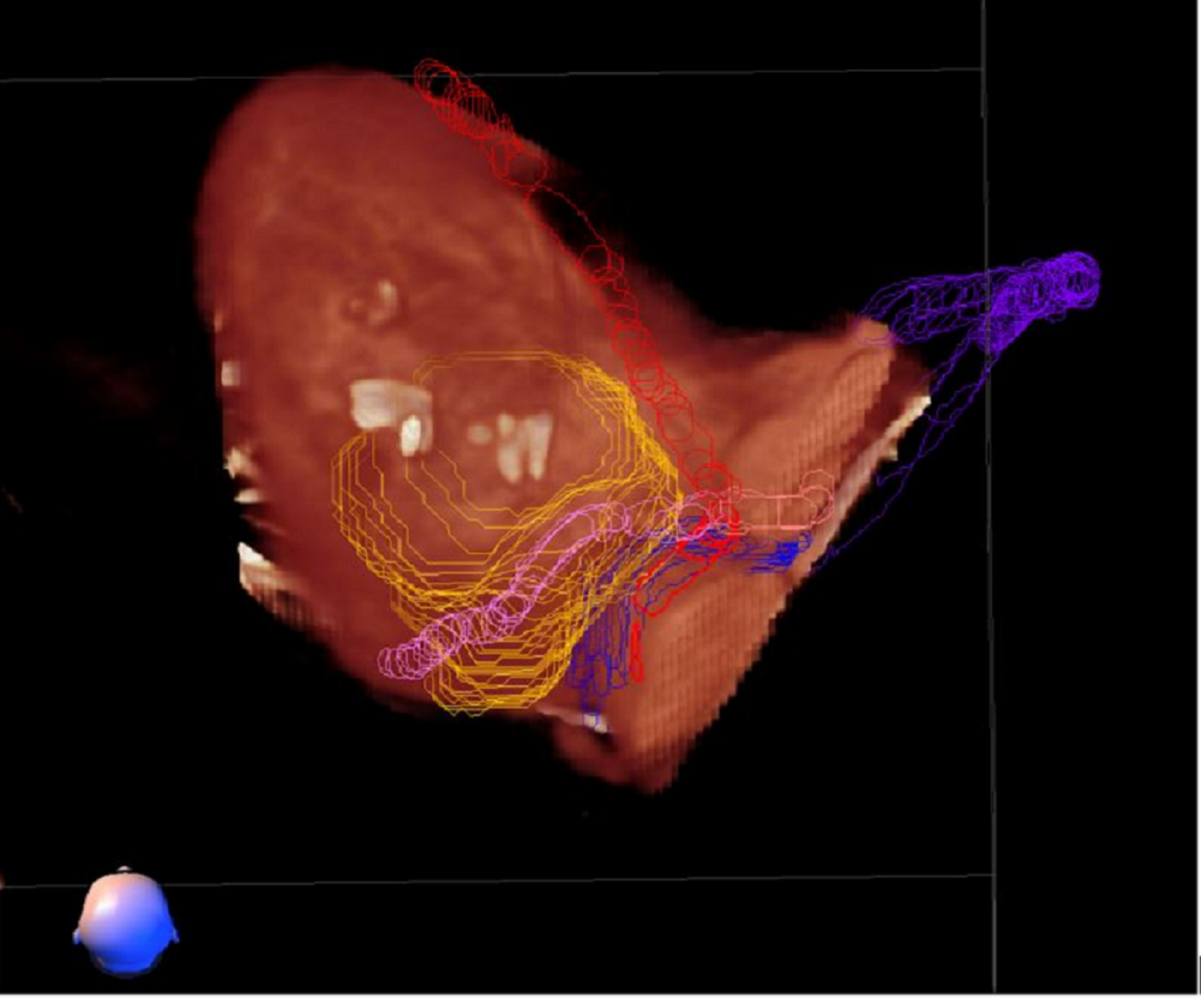
CardioPlan™ patient contouring image demonstrating the yellow target created by cardiologists, on either side of the proximal coronary arteries. Contours are sent to the treatment planning system via a DICOM file. DICOM: digital imaging and communications in medicine

All plans utilized the same contouring data set, which was originally generated for the first CyberKnife plan with a dose of 25 Gy at 6 MV photon energy prescribed for a single fraction. The CyberKnife target contours were defined by the cardiologist using proprietary software, CardioPlan 1.1, by the defining of ablation lines on the surface rendered cardiac ventricle (Figure 1). The imaging and target structure data were then exported to the MIM application to derive a contiguous clinical target volume using a cardiac-gated 4D-CT imaging study that encompassed the entire cardiac motion but was based on anticipated Synchrony tracking with the CyberKnife to compensate for respiratory motion. The third and fourth plans (TrueBeam, ITV, and gating) and the seventh and eighth plans (Edge, ITV, and gating) used expansions of the clinical target volume (CTV) to the planning target volume (ITV) consistent with the lack of respiratory tracking on that system. For the third and seventh plans with a non-gated ITV approach, the CTV was expanded by 4 mm in the superoinferior dimension and 3 mm in all other directions to generate an ITV while for the fourth and eighth plans, the CTV to ITV expansion with the gated VMAT Linac case was 2 mm isotropically. The CTV to ITV expansions were based on the results of previous respiratory motion studies for cardiac radiosurgery. The fifth, sixth, and ninth plans were generated without any expansions of the clinical target volume to purely demonstrate the dosimetric performance characteristics of the TrueBeam, Infinity, and Edge technologies. It should be noted that the three linear accelerator plans with no expansions are not to be considered practical or deliverable treatment plans and are included in this study only for theoretical comparison with the Accuray CyberKnife in evaluating the ability to cover the target and spare nearby critical structures. Only ITV and gated treatment plans were considered for clinical use in addition to the CyberKnife plans. The Elekta Infinity linear accelerator was not considered a viable treatment platform due to shortcomings in imaging technology.

**Figure 2:**
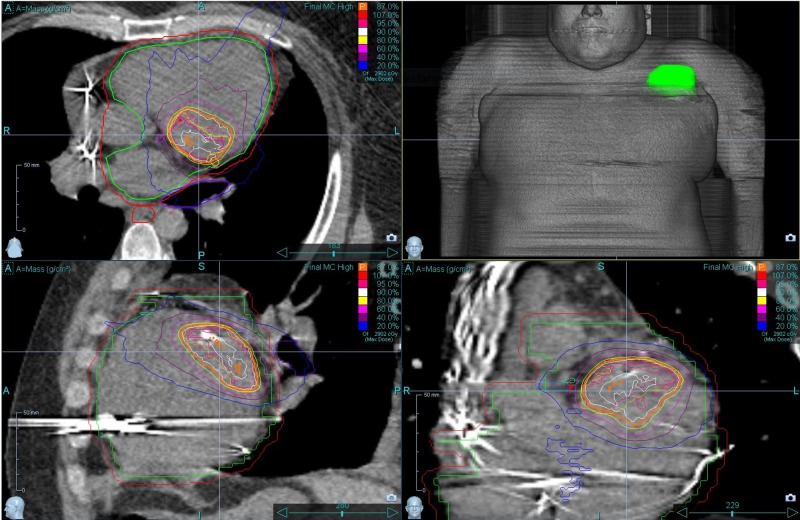
Accuray CyberKnife Dose Distribution with PTV and prescription isodose line in orange Accuray Incorporated, Sunnyvale, California, United States PTV: planning target volume

**Figure 3:**
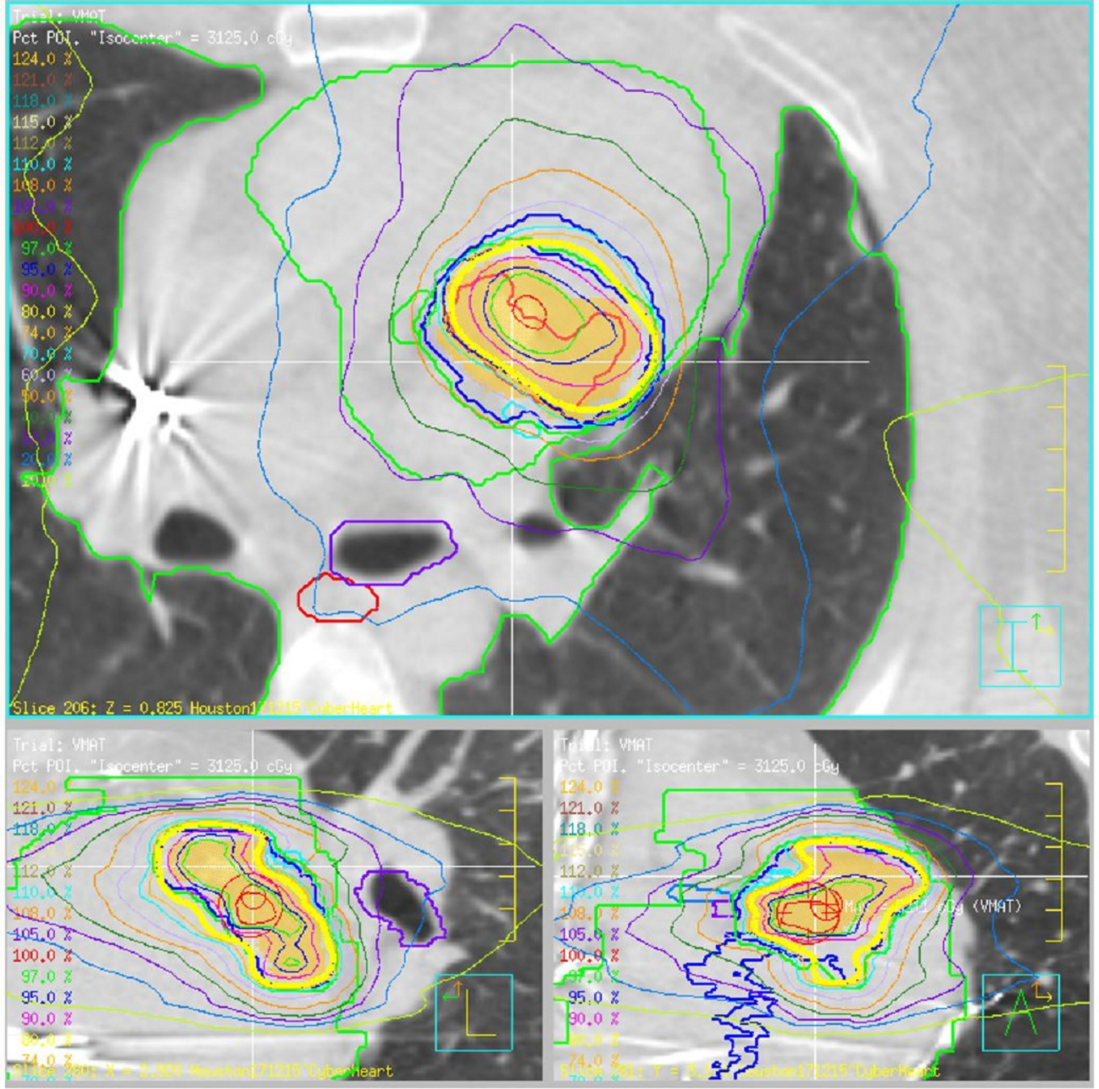
Varian TrueBeam dose distribution with PTV in orange and prescription isodose line in yellow Varian Medical Systems, Palo Alto, California, USA PTV: planning target volume

All plans utilized objective-based inverse treatment planning, sequential optimization in the case of multiplan/precision and a gradient-based simultaneous annealing optimization algorithm in the case of Pinnacle. The dose was calculated with the Raytracing and Monte Carlo dose calculation engines for the CyberKnife plans, the collapsed cone convolution algorithm for the Pinnacle treatment plans, and analytical anisotropic algorithm (AAA) with the Eclipse plans for the Edge treatment plans. The Accuray with MLC and Varian TrueBeam for gating are shown in Figure 2 and Figure 3. The treatment planning optimization objectives are listed in Table 1.

**Table 1:** Summary of optimization objectives for CyberKnife, Elekta, and Varian linear accelerator plans CyberKnife: Accuray Incorporated, Sunnyvale, California, United States; Elekta: Elekta, Stockholm, Sweden; Varian: Varian Medical Systems, Palo Alto, California, USA PTV: planning target volume; RT: right; LT: left; CA: coronary artery

Structure	Criteria	Dose Objective (Gy)
PTV	Min Dose	25.0
PTV	Min DVH D_95_	26.5
PTV	Max Dose	32.0
RT Coronary Artery	Max Dose	19.0
Ramus Coronary Artery	Max Dose	19.0
LT Anterior Descending CA	Max Dose	19.0
Mitral Valve	Max Dose	19.0
Pericardium	Max Dose	19.0
LT Main CA	Max Dose	19.0
Circumflex	Max Dose	19.0
LT Bronchus	Max Dose	20.0
Defibrillator	Max Dose	2.0

The CyberKnife plans utilized a full beam path with a single fiducial for Synchrony-based tracking. This treatment plan was based on clinical, measured beam data from St. Luke’s Hospital in Houston, TX, while the CyberKnife MLC plan utilized Accuray provided beam data. The Varian TrueBeam and Elekta Infinity plans were based on treatment machines with a 5-mm-wide leaf MLC, a single arc VMAT with an almost complete circular sweep of 340 deg and a collimator angle of 15 deg. The dose was calculated every 3 deg for this arc therapy, which resulted in 115 segments. Varian TrueBeam beam data were provided by Philips as a sample for the TrueBeam linear accelerator machine, which is considered current state-of-the-art in radiation oncology while Elekta Infinity beam data originated from the clinical practice of one of the authors. The Varian Edge plans included multiple VMAT arcs.

The optimization process for all treatment plans was implemented by balancing priorities for target coverage, dose conformity, dose homogeneity, avoidance of hot spots, and critical structure sparing. This was accomplished by assigning priorities for each of the objectives in line with the importance of the individual objectives. For the Accuray Precision CyberKnife plan, the order of objectives determined the sequence of objectives that were addressed; for the Philips Pinnacle Varian and Elekta treatment plan, priority values were assigned to each of the objectives that assigned the importance for the optimization process.

## Results

Table 2 below summarizes the administrative and logistical characteristics of the individual plans, while Table 3 shows the dosimetric comparison of the plans. For Table 3, the relevant dose volume histogram (DVH) values were extracted from the respective plans; relevance was based on clinical practice and available contours. Each treatment plan was normalized individually and due to the emphasis on dose homogeneity with Pinnacle treatment plans, the Elekta Infinity treatment plan resulted in the highest prescription isodose line. The ITV and gated Pinnacle plans on the Varian TrueBeam linear accelerators are planned to be delivered with the above-stated margins. The increased PTV size will impact the ability to spare proximal critical structures from high doses. Gating on the TrueBeam system ensures proper target coverage throughout the respiratory cycle but will increase the treatment time. If the treatment beam was gated to be active for 30% of the respiratory cycle, the treatment time would be increased by a factor of 3.3. The recorded variation in CTV volume was caused by differing peripheral voxel management methods of the different treatment planning systems. We should also acknowledge that normalization for the Accuray Precision, Varian Eclipse, and Philips Pinnacle treatment plans vary from point maximum dose to isocenter dose. That is the reason why the product of maximum dose and prescription isodose is not constant across all plans.

**Table 2:** Comparison of treatment parameters for Accuray CyberKnife, Varian TrueBeam with the ITV approach and gating, TrueBeam, Elekta no margin plans, and Varian Edge with ITV, gating, and no margin plans. The most desirable values/characteristics are in bold CyberKnife: Accuray Incorporated, Sunnyvale, California, United States; Elekta: Elekta, Stockholm, Sweden; Varian: Varian Medical Systems, Palo Alto, California, USA ITV: internal target volume; PTV: planning target volume; CTV: clinical target volume; VMAT: volumetric arc therapy; CT: computed tomography; MLC: multi-leaf collimator; CBCT: cone-beam CT; CCC: collapsed cone convolution; AAA: analytical anisotropic algorithm

Criteria/ Technology	Accuray CyberKnife w/ fixed collimator	Accuray CyberKnife w/ MLC	Varian TrueBeam (ITV)	Varian TrueBeam (Gated)	Varian TrueBeam (no expansion)	Elekta Infinity (no exp)	Varian Edge (ITV)	Varian Edge (Gated)	Varian Edge (no expansion)
Rx Dose (Gy)	25	25	25	25	25	25	25	25	25
Energy (MV)	6	6	6	6	6	6	6 FFF	6 FFF	6 FFF
Dose Rate (MU/min)	1000	1000	600	600	600	600	1400	1400	1400
Maximum Dose (Gy)	31.25	28.74	34.71	34.15	32.12	30.74	32.47	32.09	31.39
CT slice spacing (mm)	1.25	1.25	1.25	1.25	1.25	1.25	1.25	1.25	1.25
CTV/PTV (ccm)	19.80/32.55	19.69/31.79	19.80/63.25	19.80/51.45	19.80/32.55	19.80/32.55	19.4/59.4	19.4/50.0	19.40/31.5
Rx Isodose Line (%)	80	87	74	74	80	89	77	78	80
PTV coverage (%)	95.7	95.8	91.0	92.6	93.2	93.1	95	95	95
CI/nCI	1.64/1.72	1.37/1.44	1.59/1.72	1.56/1.69	1.44/1.57	1.31/1.43	0.99/1.10	0.98/1.08	0.97/1.07
Treatment Modality	Non-isocentric, non-coplanar, robotic	Non-isocentric, non-coplanar, robotic	VMAT	VMAT	VMAT	VMAT	VMAT	VMAT	VMAT
Tracking/Gating	Synchrony Tracking	Synchrony Tracking	Neither	Gating	Neither	Neither	Neither	Gating	Neither
Collimator	Fixed Collimator	2-3mm MLC	5mm MLC	5 mm MLC	5 mm MLC	5 mm MLC	2.5 mm MLC	2.5 mm MLC	2.5 mm MLC
Beam Direction	Full-Path, ~2 Pi solid angle	Full-Path, ~2 Pi solid angle	Coplanar, continuous arc, 115 segments	Coplanar, continuous arc, 115 segments	Coplanar, continuous arc, 115 segments	Coplanar, continuous arc, 115 segments	Coplanar, 3 continuous 358 degree arcs	Coplanar, 3 continuous 358 degree arcs	Coplanar, 3 continuous 358 degree arcs
Image Guidance	Orthogonal, planar, oblique, kV, intra-fraction	Orthogonal, planar, oblique, kV, intra-fraction	CBCT, pre-fraction	CBCT pre-fraction	CBCT, pre-fraction	CBCT, pre-fraction	CBCT, pre-fraction	CBCT, pre-fraction	CBCT, pre-fraction
Monitor Units	25187.8	11725.6	6767.4	6704.8	6400.7	7598.2	11511	12054	11790
Estimated Tx Time (w/o gating) (min)	95	34	9.0	29.7	9.5	11.3	8.2	8.6	8.4
Dose Calc Algorithm	Raytracing	Pencil Beam	CCC	CCC	CCC	CCC	AAA	AAA	AAA

**Table 3:** Dosimetric comparison of Accuray CyberKnife, Varian TrueBeam with ITV and gating approach, TrueBeam, Elekta no margin plans, Varian Edge with ITV, gating, and no margin plans – most desirable values are in bold. Dose maximum was reported as D0.001 ccm. CyberKnife: Accuray Incorporated, Sunnyvale, California, United States; Elekta: Elekta, Stockholm, Sweden; Varian: Varian Medical Systems, Palo Alto, California, USA ITV: internal target volume; PTV: planning target volume; CTV: clinical target volume; CT: computed tomography; MLC: multi-leaf collimator; RCA: right coronary artery; CA: coronary artery; LAD: Left anterior descending

Anatomical Structure	Accuray CyberKnife w/ cones	Accuray CyberKnife w/ MLC	Varian TrueBeam (ITV)	Varian TrueBeam (Gated)	Varian TrueBeam (No expansion)	Elekta Infinity (No expansion)	Varian Edge (ITV)	Varian Edge (Gated)	Varian Edge (No expansion)
PTV mean (Gy)	28.31	27.18	30.41	30.26	28.85	27.85	28.17	27.84	27.22
RCA max (Gy)	12.24	2.98	7.15	7.27	6.11	8.91	9.61	9.37	8.03
Ramus CA max (Gy)	21.32	28.12	25.56	24.59	22.10	21.76	24.88	24.22	20.23
LAD CA max (Gy)	21.23	21.88	24.86	23.71	22.09	20.93	24.08	22.76	18.95
L Main CA max (Gy)	14.63	13.61	22.78	21.65	19.57	18.11	14.79	13.86	11.77
Circ max (Gy)	21.35	23.18	24.18	23.11	21.07	21.06	24.26	23.08	20.36
Mitral Valve max (Gy)	19.26	22.18	29.62	28.39	25.46	24.07	25.86	25.11	21.62
Pericardium max (Gy)	31.25	28.74	34.71	34.15	32.05	30.71	32.36	31.90	31.21
Full myocar CTV mean (Gy)	31.25	27.36	31.32	31.15	32.05	30.71	29.09	28.59	27.71
Esophagus max (Gy)	5.21	4.14	10.48	9.62	8.02	10.89	4.47	4.16	3.53
Left bronchus max (Gy)	11.21	12.69	19.40	18.17	15.44	16.54	13.64	12.42	10.80
Heart max (Gy)	31.25	28.74	34.71	34.15	32.04	30.71	32.36	31.90	31.21
Heart mean (Gy)	6.61	5.25	9.08	8.14	6.58	6.01	5.77	5.20	4.07
Total Lung mean (Gy)	1.62	1.45	2.51	2.16	1.79	1.62	1.69	1.53	1.19
Cord max (Gy)	2.50	2.10	5.91	6.50	4.91	7.59	2.40	2.12	1.96
Defib max (Gy)	1.05	0.09	0.51	0.43	0.36	0.48	0.30	0.27	0.21

## Discussion

The target dose coverage and dose homogeneity are superior for the CyberKnife. Both CyberKnife plans exhibit a steeper dose gradient at the interface between target volumes and critical structures, compared to the linear accelerator plans, except for the Varian Edge treatment plans. Distant critical structures are better spared by all linear accelerator and the CyberKnife MLC plans due to the smaller amount of monitor units used and the smaller total amount of total leakage radiation reaching the patient.

Due to the use of the MLC, the smaller amount of monitor units used, and the minimization of treatment segments, all linear accelerator plans are more time-efficient in their treatment delivery compared to both Accuray CyberKnife treatment plans. A smaller treatment time is always desirable, as it minimizes involuntary patient and target movements.

As the gating and ITV approaches with the Varian TrueBeam and Varian Edge linear accelerator plans require an increased target margin, a larger volume of nearby uninvolved healthy tissues are being irradiated and nearby critical structures cannot be spared as easily as with the CyberKnife plans.

As both CyberKnife and linear accelerators are limited to 0.1% of the primary radiation to be delivered to the entire patient's body and the number of monitor units delivered by linear accelerator treatments is generally significantly lower compared to CyberKnife treatments, the whole body dose is expected to be significantly lower for all linear-accelerator-based treatment modalities. This effect is expected to decrease the secondary malignancy incidence rate due to radiation received by the patient outside of the treatment fields.

The MLC-based CyberKnife plan shows similar superior dosimetric results as the CyberKnife plan using fixed collimators with a significant improvement in treatment efficiency. The MLC usage with the CyberKnife for VT treatments should be regarded as a true improvement in treatment efficiency and a decrease in the whole body dose compared to the fixed collimator CyberKnife treatments.

## Conclusions

With real-time imaging and Synchrony-based dynamic target tracking, the CyberKnife appears to be the best-suited instrument to deliver a dose to the complex-shaped target volume in the basal region with an aberrant focus and with a nearby critical structure and the complex pulmonary and cardiac motions with superimposed frequencies and differing amplitudes. No other target locations were investigated at this point, but it is planned to cover additional locations, such as apical locations and scar lesions. It is expected that target volumes in the apical region require a larger margin for the ITV and gated treatment approaches due to the larger amplitude of target cardiac motion and, therefore, should be considered disadvantaged compared to the CyberKnife treatment with tracking capabilities due to their inability to spare nearby critical structures. Also, the treatment efficiency for gated treatments is expected to decrease due to a more erratic target motion and fewer cardiac phase angles that can be utilized for the "Beam On" phase during the gating process.

From a dosimetric evaluation perspective, it can be concluded that the CyberKnife provides better sparing for critical anatomical structures in close proximity to the target while anatomies at several centimeters distance are better spared by Linac-based techniques. This is caused by the ability of the CyberKnife to produce very steep dose gradients in the immediate vicinity of the target volume and the fact that many more monitor units are delivered for the CyberKnife as compared to the Linac, resulting in a larger amount of leakage radiation reaching the more distant structures.

The differences in dose conformity are apparent in comparison to the CyberKnife with the no expansion TrueBeam and Elekta cases but they are more pronounced for the gated and ITV cases. As the PTV volumes increase for the gated TrueBeam and TrueBeam ITV compared to the CyberKnife, it is, in principle, increasingly more difficult to cover the target volumes, spare the nearby critical structures, and conform the prescription isodose line to the target.

Overall, for this basal target volume, the CyberKnife should be considered the more precise delivery technology for VT treatments, as it can provide real-time tracking, intra-fraction imaging, more accurate dose painting, and better sparing of nearby critical anatomical structures. However, the cost of this achievement remains poor treatment efficiency, which is overcome partially with the employment of the MLC for the CyberKnife treatment.
